# A Small Short-Necked Hupehsuchian from the Lower Triassic of Hubei Province, China

**DOI:** 10.1371/journal.pone.0115244

**Published:** 2014-12-17

**Authors:** Xiao-hong Chen, Ryosuke Motani, Long Cheng, Da-yong Jiang, Olivier Rieppel

**Affiliations:** 1 Wuhan Centre of China Geological Survey, Wuhan, Hubei 430023, P. R. China; 2 Department of Earth and Planetary Sciences, University of California Davis, Davis, California 95616, United States of America; 3 Laboratory of Orogenic Belt and Crustal Evolution, MOE, Department of Geology and Geological Museum, Peking University, Yiheyuan Str. 5, Beijing 100871, P.R. China; 4 Center of Integrative Research, The Field Museum, Chicago, Illinois 60605–2496, United States of America; Raymond M. Alf Museum of Paleontology, United States of America

## Abstract

Hupehsuchia is a group of enigmatic Triassic marine reptiles that is known exclusively from two counties in Hubei Province, China. One of the common features of the group was a modestly long neck with nine to ten cervical vertebrae. We report a new species of Hupehsuchia, *Eohupehsuchus brevicollis* gen. et sp. nov., which for the first time shows a short neck in this group, with six cervicals. The configuration of the skull roof in *Eohupehsuchus* is also unique among Hupehsuchia, with narrow frontals and posteriorly shifted parietals, warranting recognition of a new species. The taxon superficially resembles *Nanchangosaurus* in retaining hupehsuchian plesiomorphies, such as low neural spines and small body size. However, its limbs are well-developed, unlike in *Nanchangosaurus*, although the latter genus is marginally larger in body length. Thus, the individual is unlikely to be immature. Also, *Eohupehsuchus* shares a suite of synapomorphies with *Hupehsuchus*, including the second and third layers of dermal ossicles above the dorsal neural spines. A phylogenetic analysis suggests that the new species is not the most basal hupehsuchian despite its short neck, and instead forms the sister taxon of Hupehsuchidae. Until recently, Hupehsuchia contained only two monotypic genera. Now there are at least four genera among Hupehsuchia, and the undescribed diversity is even higher. The left forelimb of the only specimen is incomplete, ending with broken phalanges distally. The breakage could only have occurred pre-burial. The individual may have been attacked by a predator and escaped, given that scavenging is unlikely.

## Introduction

The Mesozoic saw the emergence of multiple marine reptile groups including Hupehsuchia, which is only known from the Spathian (Lower Triassic) of Hubei Province, China [1]. Hupehsuchians have been known to science for more than half a century since the discovery of *Nanchangosaurus suni* Wang, 1959 [2], although the name Hupehsuchia was not coined until *Hupehsuchus nanchangensis* was described by Young in 1972 [3]. Until recently, their anatomy and diversity have been poorly understood despite the notable pioneering work by previous authors [2]–[5]. Only two monotypic genera and a third unnamed genus represented by an impression fossil (IVPP V4070, Institute of Vertebrate Paleontology and Paleoanthropology, Academia Sinica) [4] were known, and none of the specimens were well-preserved. This left their affinities within Diapsida ambiguous, although a close relationship with ichthyosaurs was suspected [4], [6].

The level of knowledge has significantly improved recently. Ongoing fieldwork by the Wuhan Centre of China Geological Survey (WGSC) has yielded new hupehsuchian specimens from Nanzhang and Yuan'an Counties, Hubei Province, China. So far, the specimens allowed us to improve knowledge of the anatomy of the basal hupehsuchian *Nanchangosaurus* [7] and also to describe a new genus, *Parahupehsuchus*, whose peculiar trunk contained a nearly complete tube of bones [8]. In addition, the suggested phylogenetic affinities with ichthyosaurs [4] are now strongly supported based on cladistic analyses [7].

Hupehsuchians typically have a heavily ossified body trunk with pachyostotic ribs and gastralia that overlap adjacent elements, limiting its flexibility [8]. The neural spines are bipartite across a large part of the trunk, and dermal ossicles are present above the neural spines. The snout is dorso-ventrally flattened, elongated, and edentulous. The peculiar combination of features have made the lifestyles of Hupehsuchia ambiguous [4].

Despite such progress, our knowledge of Hupehsuchia is far from complete. The present paper reports a new hupehsuchian discovered in the course of fieldwork conducted by WGSC.

## Materials and Methods

### Specimen

The main specimen for the present study is WGSC (Wuhan Centre of Geological Survey, China) V26003, representing a new species as described below. It was collected during field excavation in 2011 in Yuan'an County, Hubei Province, China. The specimen was excavated with the proper permit from the Bureau of Land and Resources, China, and is accessioned in the fossil collection at the central facility of WGSC in Wuhan, Hubei Province, China.

### Phylogenetic Analysis

We expanded a published data matrix [8] by adding the new species and 11 new characters. Additionally, one of the original characters was recoded as two separate characters (character 14 of [8] was divided into new characters 18 and 19), while five others were removed because they were found parsimony informative although they might be useful in identifying the basal synapomorphies of Ichthyosauromorpha (characters 4, 5, 6, 7 and 22, of [8]). *Cartorhynchus* replaced *Utatsusaurus*, reflecting the latest finding of this most basal ichthyopterygian [9]. As a result, the matrix now contains 32 discrete osteological characters coded for three outgroup and five ingroup taxa. The data matrix and descriptions of the characters are found in S1 Text.

We analyzed the resulting data matrix using PAUP* 4b10 (Phylogenetic Analysis Using Parsimony) [10]. A branch and bound search, which ensures discovery of all most parsimonious trees, was implemented given the small data matrix. The result was confirmed by an ienum search in TNT 1.1 [11], which was also used to calculate the Bremer index and bootstrap (n = 1000) values.

#### Measurements

Small structures below 170 mm were measured using Mitutoyo digital calipers that displays the length down to 0.01 mm. Larger structures were measured using a metal tape measure, and recorded to the nearest mm.

#### Nomenclatural Acts

The electronic edition of this article conforms to the requirements of the amended International Code of Zoological Nomenclature, and hence the new names contained herein are available under that Code from the electronic edition of this article. This published work and the nomenclatural acts it contains have been registered in ZooBank, the online registration system for the ICZN. The ZooBank LSIDs (Life Science Identifiers) can be resolved and the associated information viewed through any standard web browser by appending the LSID to the prefix “http://zoobank.org/”. The LSID for this publication is: urn:lsid:zoobank.org:pub:1B8887A9-F513-427D-BE9A-DBBB15DD9A44. The electronic edition of this work was published in a journal with an ISSN, and has been archived and is available from the following digital repositories: PubMed Central, LOCKSS.

## Results

### Systematic Paleontology

#### Systematic hierarchy

Reptilia Laurenti 1768 [12]

Diapsida Osborn 1903 [13]

Hupehsuchia Young 1972 [3]

*Eohupehsuchus brevicollis* gen. et sp. nov. urn:lsid:zoobank.org:act:94F905DD-8995-4477-841A-85DEDA8C8287

#### Etymology

Generic name combines εοσ (Gr. early, dawn), hupeh (alternative spelling of Hubei Province), and Σοũχος (Gr. name for the Egyptian crocodile deity Sobek). Specific name is a combination of brevis (L. short) and collum (L. neck), referring to the unusually short neck for a hupehsuchian.

#### Holotype

**Figure 1:**
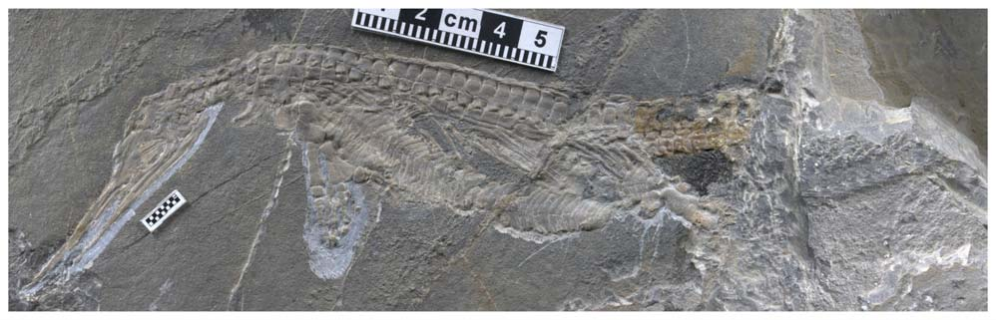
The holotype of *Eohupehsuchus brevicollis*, WGSC V26003. Scales are in centimeters.

**Figure 2:**
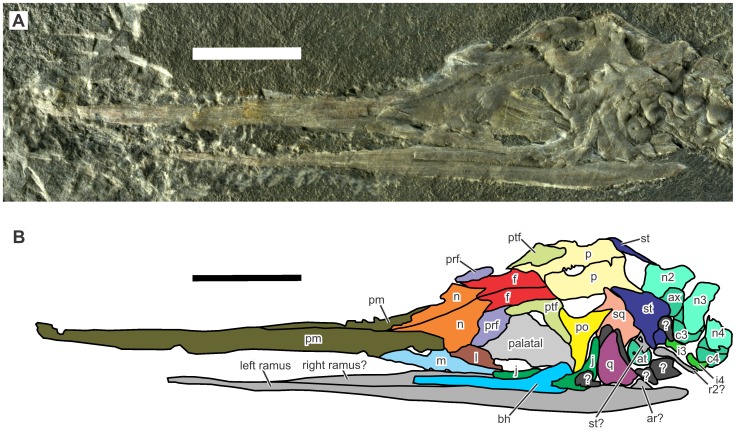
Skull of *Eohupehsuchus brevicollis*, WGSC V26003. Scales are 1 cm long. Symbols: ar, articular; at, atlantal pleurocentrum; ax, axis centrum; bh, basihyal (?); c#, centrum; f, frontal; i#, intercentrum; j, jugal; l, lacrimal; m, maxilla; n, nasal; n#, neural spines; p, parietal; pm, premaxilla; po, postorbital; prf, prefrontal; ptf, postfrontal; q, quadrate; r#, rib; sq, squamosal, st, supratemporal. Colors: dark gray, unidentified bones; light gray, unidentified palatal bones; medium gray, unidentified mandibular bones.

**Figure 3:**
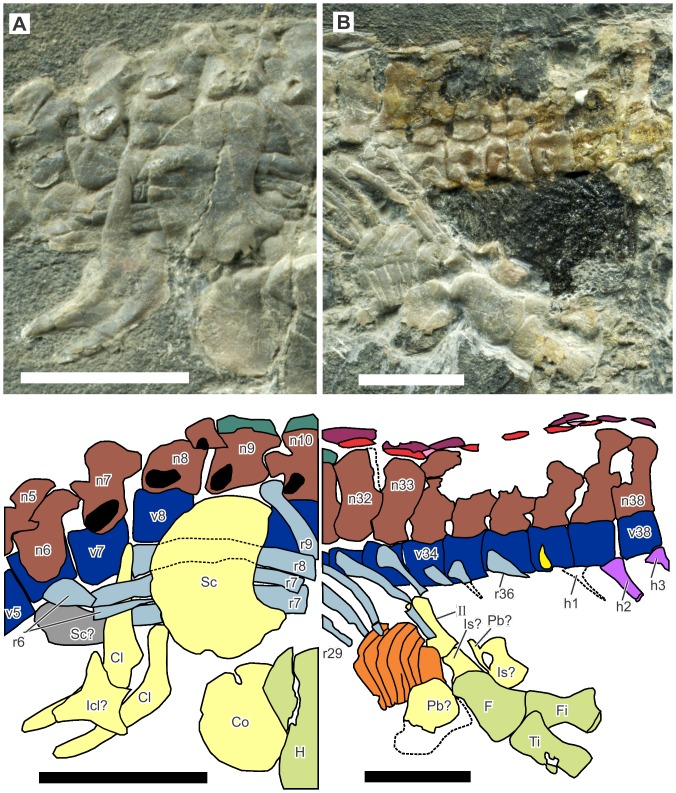
Pectoral and pelvic regions of *Eohupehsuchus brevicollis*, WGSC V26003. A, pectoral region. B, pelvic region. Symbols: Cl, clavicle; Co, coracoid; F, femur; Fi, fibula; H, humerus; h#, hemal spine; Icl, interclavicle; Il, ilium; Is, ischium; n#, neural spine; Pb, pubis; r#, rib; Sc, scapula; v# vertebral centrum. Colors: blue, vertebral centra; brown, neural spine first segment; green, neural spine second segment; light blue, rib; light green, limb elements; light purple, hemal spines; light yellow, girdle elements; orange, gastral elements; pink, dermal armor second layer; red, dermal armor first layer; red-purple, dermal armor third layer; yellow, parapophysis. Scale bars are 1 cm long.

**Figure 4:**
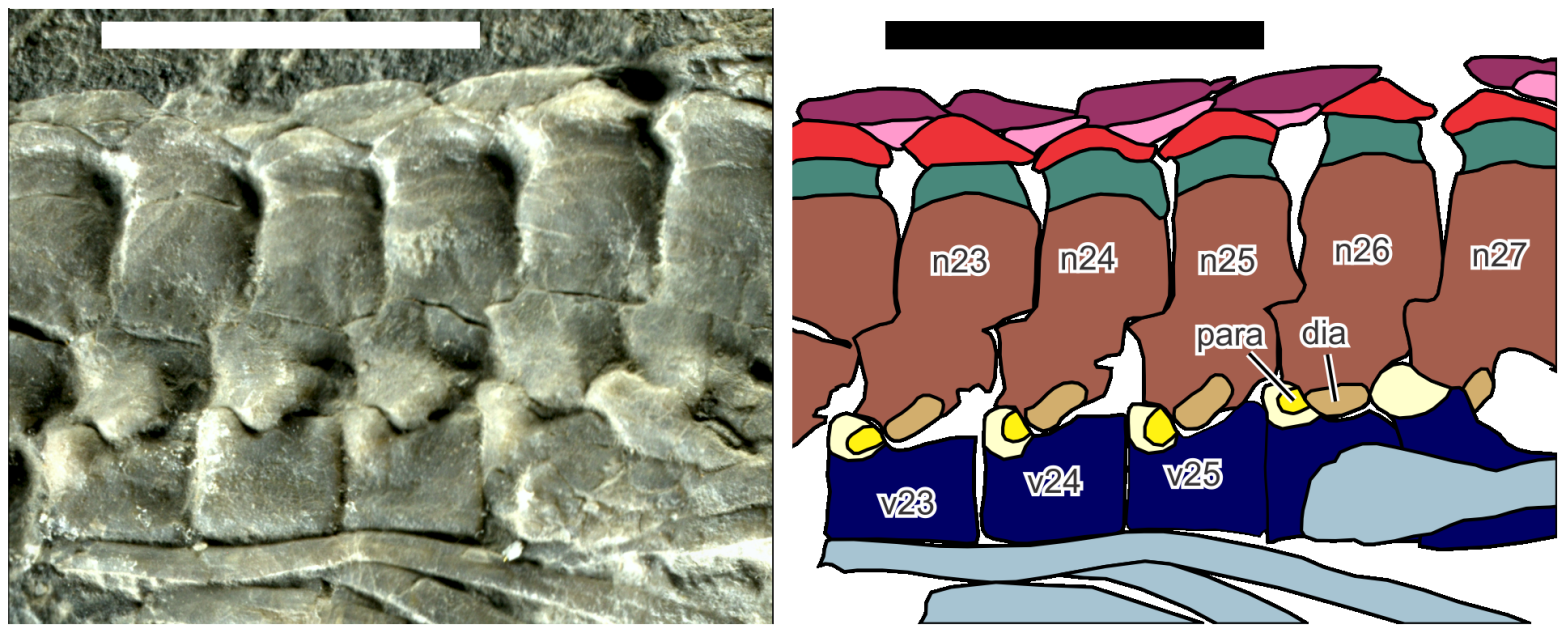
Mid-dorsal vertebrae of *Eohupehsuchus brevicollis*, WGSC V26003. Colors and symbols are the same as in Fig. 3 except: light brown, diapophysis (dia); light yellow, swelling surrounding parapophysis; and yellow, parapophysis (para). Scale bars are 1 cm long.

**Figure 5:**
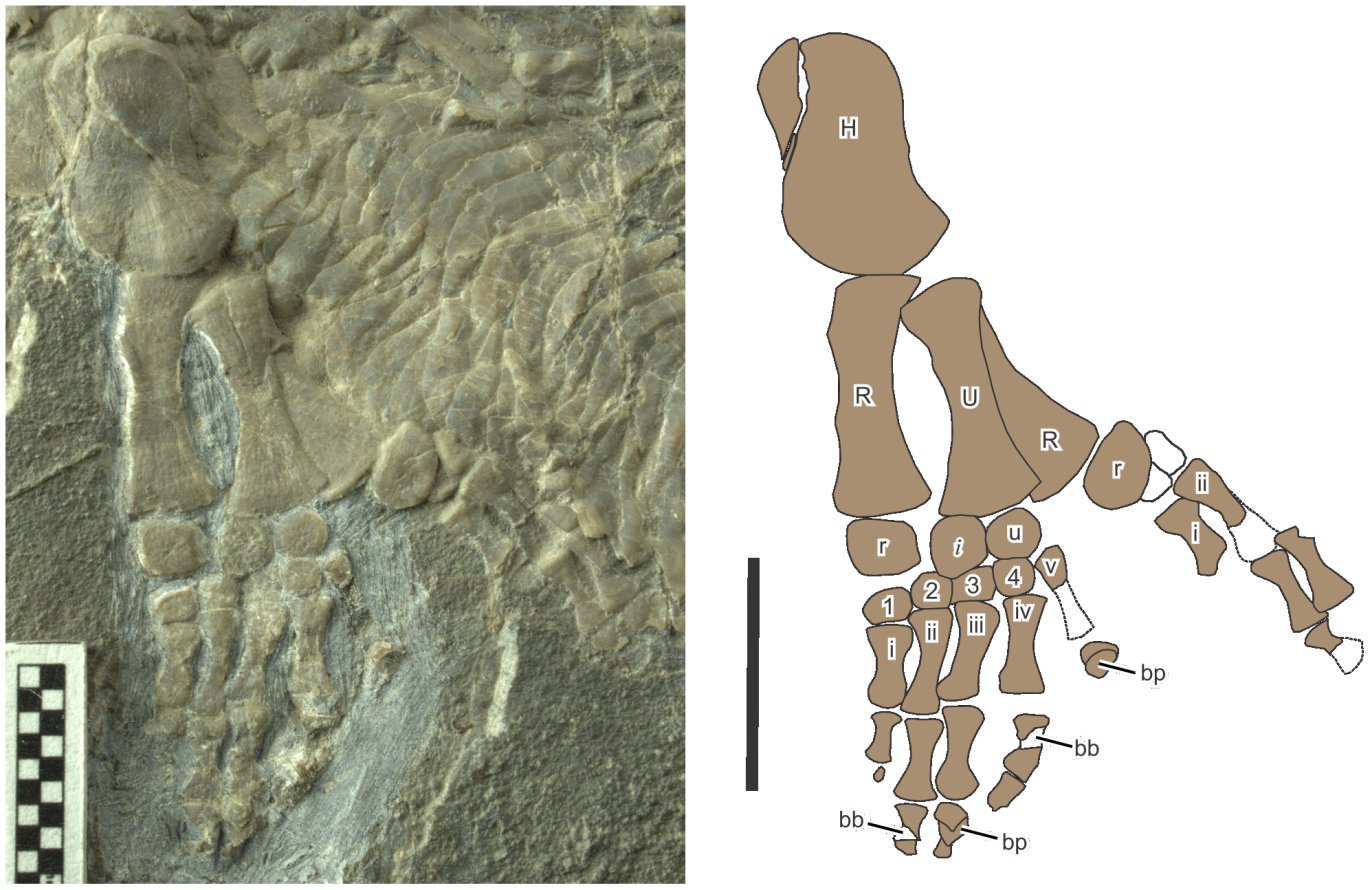
Forelimbs of *Eohupehsuchus brevicollis*, WGSC V26003. Symbols: bb, broken bones that are kinked from damage; bp, broken bone pieces that are dislocated; *i*, intermedium; i–v, metacarpal; H, humerus, R, radius; r, radiale; U, ulna; u, ulnare; 1–5, distal carpal. Scales are 1 cm long.

WGSC 26003 (Figs. 1–5).

#### Diagnosis

(Autapomorphies) Frontal not widened rostrally or caudally; parietal posteriorly located, with rostral margin in level with rostral margin of upper temporal fenestra; pineal foramen located posteriorly relative to the rostral margin of upper temporal fenestra; neck short, with six cervical vertebrae; interclavicle with long cranial process; third-layer dermal element short, at about one vertebral-segment long; dermal armor elements very thin.

#### Locality and Horizon

Upper Spathian (Lower Triassic) Jialingjiang Formation, exposed in Yangping, Yuan'an County, Hubei Province, China [14].

### Description

Selected measurements of the holotype are given in Table 1. The preserved length of the specimen is 23.6 cm, of which 17.4 cm is precaudal. The total length would be about 40 cm, assuming the body proportion of *Hupehsuchus*. Therefore, this is the smallest hupehsuchian known so far. Note, however, that *Nanchangosaurus*, with precaudal lengths slightly greater than 20 cm [7], is similarly small. A large part of the skeleton is re-crystallized and somewhat translucent under a strong light. The specimen was found exposed on the surface and consequently suffered from erosion to various extents. Parts of several bones, such as the premaxilla, dentary, left radius and radiale, are missing because of erosion.

**Table 1:** Selected measurements from WGSC 26003 in mm.

Preserved Length	236
Preserved Precaudal Length	174
Preserved Skull Length	56.51
Orbit Length	8.11
Orbit Height	5.41
Upper Temporal Fenestra Length	2.89
Retroarticular Process Length	4.26
Scapula Maximum Length	9.34
Scapula Proximal Width	6.22
Clavicle Length	12.42
Clavicle Maximum Width	2.19
Humerus Length	10.64
Humerus Proximal Width	6.07
Humerus Distal Width	6.92
Radius Length	10.38
Radius Proximal Width	2.84
Radius Distal Width	3.99
Ulna Length	10.19
Ulna Proximal Width	3.60
Ulna Distal Width	4.31
Last Dorsal Centrum Length	4.04
2nd Caudal Centrum Length	3.72

#### Cranium

The preserved skull length is 56.51 mm, without including the tips of the premaxillae, which are missing because of erosion. The skull of *Eohupehsuchus* (Fig. 2) closely resembles that of *Nanchangosaurus* and *Hupehsuchus*. The edentulous premaxilla is long, extending over more than half of the total skull length. The lateral exposure of maxilla is small, although the palatal extension of the bone may be larger. The maxilla has a short post-narial process that ascends from the main body to separate the lacrimal from the external naris. The nasal is wide but not very long, not extending rostrally very much beyond the external naris.

The orbit is 8.11 mm long, smaller than 10.84 mm in *Nanchangosaurus* (WGSC V26006)—thus, *Eohupehsuchus* has a smaller relative eye size than *Nanchangosaurus*. The margin of the orbit is defined by the lacrimal, prefrontal, postfrontal, postorbital and jugal, without the frontal entering the dorsal margin of the orbit, at least on the left side of the skull. However, this may be a preservational artifact given the lateral exposure of the specimen, as discussed by [7]. The lacrimal forms a band between the maxilla and prefrontal, which seems to overlap the dorsal part of the lacrimal laterally. The prefrontal is thickened posteriorly, near the base of a fan-shaped eyebrow that overhangs the rostro-dorsal corner of the orbit. Similar thickening is known in the type specimen of *Nanchangosaurus suni*.

The cheek is open ventrally, with a narrow incision representing the lower temporal fenestra. The relevant region of the specimen is slightly disarticulated. The jugal is J-shaped without a quadratojugal process to close the lower temporal fenestra ventrally. It has been broken into two pieces, probably because compaction pushed the bone against the underlying hyoid body, which is thick and robust. The caudal piece of the jugal is dislocated toward the quadrate, causing the bone to disarticulate from the postorbital and narrowly touch the squamosal. The dislocation also contributed to postmortem closure of the lower temporal fenestra. The quadrate has also been displaced but toward the rostrum, as is evident from its anterior location compared to its socket at the conjunction between the squamosal and supratemporal. This dislocation, again, contributed to the postmortem closure of the cheek. The quadratojugal is not preserved, although it may have been present in life as in *Nanchangosaurus* and *Hupehsuchus*.

The frontal is approximately rectangular, with a forked but not widened rostral end. This forking is in common with *Nanchangosaurus* and *Hupehsuchus*. However, the overall shape of the frontal appears different from that seen in the latter two genera, because a rostral and caudal widening of the bone, as well as a tapering caudo-lateral process, is absent in *Eohupehsuchus*. The absence of the process is related to the overall posterior location of the parietal, which overlies the frontal with a medial rostral process in the two genera. A large pineal foramen is enclosed by the two parietals, and the foramen is located within the anterior halves of the bones. The rostral margin of the pineal foramen lies well posterior to the rostral extent of the upper temporal fenestra in *Eohupehsuchus*, again because of the posterior positioning of the parietal—the anterior extents of the two temporal fenestrae are sub-equal or the pineal foramen is slightly more rostral than the upper temporal fenestra in the other two genera. The upper temporal fenestra, bordered by the parietal, squamosal, postorbital, and prefrontal, is small. The squamosal excludes the supratemporal from the margin of the temporal fenestra, as in *Nanchangosaurus* and *Hupehsuchus*—this strange arrangement therefore seems common among hupehsuchians. The supratemporal forms a ‘lappet’ toward the occiput, as in other hupehsuchians.

#### Axial skeleton

The specimen preserves six cervical, 26 dorsal, at least one sacral, and at least 14 caudal vertebrae. The posterior limit of the cervical region was judged based on rib morphology. The clavicle extends anteriorly to the level of the sixth cervical centrum but the first rib that is clearly elongated is associated with the seventh vertebra, which is identified as the first dorsal vertebra (Fig. 3A). The 33rd vertebra was identified as a sacral based on its associated rib that is short, slightly expanded distally, and closely associated with the ilium (Fig. 3B). The 34th vertebra may also be sacral but its broken rib prevents us from judging its identity. The dorsal count of *Eohupehsuchus* is similar to the counts of 26 to 27 in *Nanchangosaurus* [7]. However, the cervical count is significantly different between the two genera, with 10 cervicals in *Nanchangosaurus* versus six in *Eohupehsuchus*. As a result, there are only 32 presacral vertebrae in *Eohupehsuchus*, in contrast to 36 to 37 in *Nanchangosaurus* and 37 to 38 in *Hupehsuchus* [7]. Five to six caudal vertebrae and a neural spine are preserved posteriorly in the main string of vertebrae. In addition, a second string is dislocated postero-ventrally, containing eight additional caudal vertebrae (Fig. 1). It is unknown how many are missing in between the two strings of vertebrae.

The parapophysis is clearly present in posterior dorsal vertebrae (Fig. 4), joining the diapophysis of the neural arch to form the synapophysis as in *Parahupehsuchus*. However, the parapophysis lacks a second articular facets for the peculiar rib articulation seen in the latter genus. The synapophysis is inclined relative to the centrum, at about 30°. The angle is shallow but not as much so as in *Parahupehsuchus*. The synapophysis is not present in *Hupehsuchus*, and probably not in *Nanchangosaurus*. The articulation between the parapophysis and diapophysis is at about 45° to the horizontal plane of the centrum, i.e., the diapophysis descends to the level of the parapophysis and articulates with the caudo-dorsal side of the latter. The parapophysis forms a cranio-dorsal blob on the centrum, which is distinctly swollen. Anterior dorsal vertebrae are largely displaced, covered, or eroded, making it difficult to see the parapophysis. However, the most craniad vertebra to clearly show the parapophysis is the eighth dorsal vertebra, in which the centrum is dorsally exposed, revealing cranio-dorsal swellings corresponding to the parapophysis. The first three dorsal vertebrae seem to lack the parapophysis—therefore, the rib articulates solely with the diapophysis in this region. A reversed transition occurs posteriorly. The second sacral neural spine is the last in the series to have a diapophysis, whereas the first caudal lacks the structure—therefore, the caudal ribs articulate only with the parapophysis. The parapophysis exists in the first four caudal vertebrae but not on the fifth and thereafter. Hemal spines appear at the fifth caudal vertebra—the first hemal spine was damaged during preparation but its impression is still present between the fourth and fifth caudal vertebrae.

Each dorsal rib bears a longitudinal groove, as in the condition in *Nanchangosaurus* but unlike the condition in *Hupehsuchus* or *Parahupehsuchus*. The erosion of the specimen is such that the proximal parts of the ribs are rarely preserved. There is a rib that seems to articulate with the 26th vertebra, i.e., the 20th dorsal rib. The proximal head of the rib is complete and carries a small posterior flange proximally (Fig. 4), as expected in hupehsuchians.

The neural arches show an approximately constant height throughout the posterior cervical to the anteriormost caudal region. In contrast, the neural spines change their height through the vertebral series, although none of them is very tall. They are very low anteriorly, with neural arches taller than the corresponding dorsal neural spine. The neural arch and spine become sub-equal in height in the anterior dorsal region, near the eighth dorsal vertebra. More posteriorly, the neural spines are taller than the arches, and the tallest neural spines are found near the pelvic region, where they are about twice as tall as the neural arch. The second segment of the neural spines first appear in the pectoral region, in the third dorsal vertebra. From there, it exists throughout the dorsal series but disappears in the sacral neural spines. Caudal neural spines are very poorly preserved, so it is impossible to judge the presence of the second segment.

The first layer of dermal ossicles lies above the neural spines, one ossicle per spine, as in other hupehsuchians. The first ossicle appears above the first dorsal vertebra, although it is small and not fused to the neural arch. Where the second segment of neural spine appears in the third dorsal vertebra, the ossicle starts to fuse to this segment. The first layer ossicles persist into the anterior caudal series, at least down to the seventh caudal vertebra.

A second and third layer of dermal ossicles occurs in *Eohupehsuchus* (Fig. 4) unlike in *Nanchangosaurus*. The first second-layer element lies cranio-dorsal to the ninth dorsal vertebra, whereas the first third-layer ossicle is found above the tenth and eleventh dorsal vertebrae. As in *Hupehsuchus* and *Parahupehsuchus*, the second-layer elements fill the gap between the first-layer elements, whereas the third-layer ossicles are larger, spanning two to three vertebrae. The second-layer elements never touch one another, unlike the first- or third-layer ossicles (Fig. 4). The dermal ossicles are thin relative to their lengths, compared to those of *Hupehsuchus* or *Parahupehsuchus*. Also, third-layer elements are short, for being only about one vertebral-segment long. In *Hupehsuchus* and *Parahupehsuchus*, the elements are as long as about 1.5 to 2 vertebral segments.

The gastralia comprise rows of three elements, two lateral and one median. All three elements are boomerang-shaped, but the median one points caudally instead of cranially as in lateral elements. Each lateral element partly overlaps its rostral counterpart laterally, except the most cranial element. The overlap amounts to about one third of the total width of the element. The median element is smaller than the lateral ones and seems to fill the gap between the lateral elements, without touching another median element. The three elements interlock tightly to form an approximate Σ shape that is widely open. The interlocking is best preserved caudal to the left ulna, where both right and left lateral elements are exposed in ventral view, with the median element filling the gap between them. The lateral element is approximately as long as three nearby dorsal vertebral centra combined, which is similar to *Parahupehsuchus* and *Hupehsuchus* but not as long as in *Nanchangosaurus*, in which it matches the length of four centra. There are about two lateral elements per vertebral segment, although the ratio is not constant. The lateral element is symmetrical as in *Nanchangosaurus* but unlike in *Hupehsuchus* or *Parahupehsuchus*. The medial element is flattened as in *Nanchangosaurus*, whereas *Hupehsuchus* and *Parahupehsuchus* have a thick and narrow median element with round cross-section.

#### Appendicular skeleton

The coracoid of *Eohupehsuchus* is partly concealed by the humerus (Fig. 3A) but it is obvious that the bone is distinctly smaller than the scapula, as is also the case in *Parahupehsuchus* and *Nanchangosaurus* [7], [8]. The coracoid foramen exists as a notch that is open proximally, craniad to the scapular facet, as in *Parahupehsuchus* [8]. The scapula also resembles that of *Parahupehsuchus* in being fan-shaped—the bone is incompletely known in *Nanchangosaurus* and *Hupehsuchus*. The clavicle again resembles that of other hupehsuchians in having a prominent cranial process that equals the scapular process in length. The clavicle, which is approximately boomerang-shaped, has cranial and scapular processes that taper quickly and lack flanges near the two ends. The interclavicle is damaged, but it was approximately rhomboidal, with its cranial process much better developed than the caudal counterpart (Fig. 3A).

The left forelimb shows a strange preservation, the interpretation of which will be discussed later. The manus is incomplete distally (Fig. 5) but this is not because of preparation error or weathering. At the time of discovery, the last phalanges to be exposed for respective digits were at an angle with the bedding plane, appearing as if they were descending into the matrix distally. After careful preparation of the relevant region, however, it was revealed that the bones did not continue very far into the matrix. Rather, they were abruptly terminated, with uneven broken surfaces. The first phalanx of the fourth digit (phalanx iv-1) and the phalanx ii-2 are kinked around the damage mid-shaft (Fig. 5, bb). Phalanges iii-2 and v-1 are broken and their pieces overlap (Fig. 5, bp); in the case of phalanx iii-2, the pieces also overlap phalanx iii-3 that has shifted proximally. Phalanx iv-2 is incomplete distally without a trace of bone splinters (Fig. 5). The breaks are approximately aligned along a line that seems parallel the general direction of geological joints in the rock, yet there is no displacement of matrix observed in this particular area. Therefore, the breakage could only have occurred before the specimen was buried.

The forelimb of *Eohupehsuchus* (Fig. 5) shares more features with that of *Hupehsuchus* than that of *Nanchangosaurus*. The extremities of limb bones are generally well developed, suggesting maturity despite of the small body size. The stylopodial and zeugopodial bones are elongated, unlike the poorly developed bones of *Nanchangosaurus* whose body size is slightly larger. The humerus has an anterior flange that is slightly concave rostrally. The recrystallization of the left radius shaft removed all surface striations. This, together with the damage along the rostral margin of the bone, prevent confirmation of the presence of an anterior flange on the bone, which is expected in hupehsuchians. The left radiale is damaged, but the right one reveals a wide bone that is proximally straight and distally rounded, a typical hupehsuchian feature. The radiale is the largest carpal element. There are three proximal carpals, namely radiale, intermedium, and ulnare, plus four distal carpals (dc). There is no dc 5, and the fifth metacarpal (mc) articulated directly with the ulnare and dc4. Mc5 is distinctively more slender than mc1-4. The phalangeal formula cannot be established because of the aforementioned damage to the forelimb distally.

The left humerus is 10.64 mm long, which is at least 20% longer than in the two known specimens of *Nanchangosaurus*. As mentioned earlier, these latter specimens are slightly larger in body size, by about 12.5%. Therefore, *Eohupehsuchus* has a relatively much larger forelimb compared to *Nanchangosaurus*.

The pelvic girdle is not well-preserved, partly because of erosion (Fig. 3B). The complete outline is not known for any of the three pelvic elements. The following description is based on what is preserved and exposed, with interpretations. The ilium is a straight bone that is about 2.5 times longer than wide. The proximal end seems to have two facets, each of which is slightly wider than the ribs. Therefore, it is expected that there are two sacral vertebrae. However, as noted earlier, poor preservation prevents confirmation of the second sacral vertebra. The pubis is plate-like, and the ischium is constricted in the middle. A better specimen is needed to clarify the morphology of the pelvic girdle.

The hind limb is known only from the three proximal elements of the left limb. The femur is much wider distally than proximally. The femoral head appears slightly dislocated caudally relative to the center-line of the bone. The tibia is wider proximally than distally. It is more slender than in *Hupehsuchus* or *Parahupehsuchus*. The fibula, which is wider distally than proximally, is deflected caudally beyond the posterior margin of the fibula, as in other hupehsuchians.

### Phylogenetic Analysis

**Figure 6:**
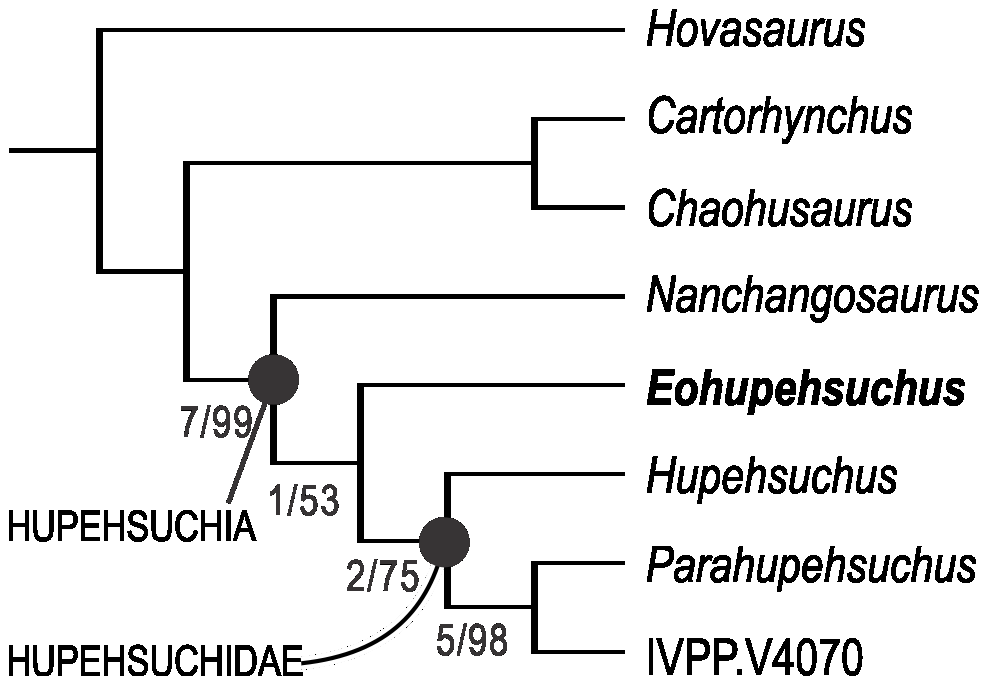
Phylogenetic hypothesis of Hupehsuchia. Numbers are Bremer index/bootstrap values (1000 replications). See text for tree statistics.

Branch and bound (called ienum in TNT) searches identified a single most parsimonious tree in both PAUP* 4b10 and TNT 1.1 (TL = 42, CI = 0.762, RI = 0.787). The small number is understandable given that there are only five ingroup taxa (Fig. 6). *Eohupehsuchus* appeared as the sister taxon of Hupehsuchidae, the clade comprising *Hupehsuchus, Parahupehsuchus* and IVPP V4070. *Nanchangosaurus* remained the most basal hupehsuchian, lying outside the clade of *Eohupehsuchus* and Hupehsuchidae.

## Discussion

It is firstly important to consider the possibility that the type and only specimen of *Eohupehsuchus brevicollis* may represent a growth stage of another taxon. If it was a growth stage of *Nanchangosaurus suni*, the body size would suggest it to be the youngest individual known for the species since the specimen is smaller than the two known specimens of *N. suni*. However, ossification of its limb is more advanced than seen in the specimens of *N*. *suni*. For example, the humerus is about 30 to 40% longer in *Eohupehsuchus* relative to body size than in *Nanchangosaurus*. Moreover, it is unlikely that the cervical count changes from six to ten through postembryonic growth—note that the dorsal counts are identical between the two taxa. It is not known if the number of dermal ossicle layers change through growth in Hupehsuchia; even if it does, it would be strange for this smallest individual to have three layers whereas the two larger specimens have only one each. Therefore, it is most likely that *E*. *brevicollis* is not a juvenile of *N*. *suni*.

*Eohupehsuchus brevicollis* is also unlikely to be a juvenile of *Hupehsuchus nanchangensis*, whose specimens are about twice as large. First, its cervical and dorsal counts (6 and 26, respectively) both differ from those for *H. nanchangensis* (9–10 and 28, respectively). These vertebral counts are not expected to increase during postembryonic growth. Second, the surface of the left humerus in WGSC V26003 appears to be well-finished, suggesting that the individual was mature. Similarly, extremities of the autopodial elements are well-developed, and carpals are well-packed, again indicating that the individual was osteologically mature. Third, the fusion between the first and second segment of neural spines is at least as progressed as in *Hupehsuchus nanchangensis*.

The short neck of *Eohupehsuchus* is unusual among Hupehsuchia, whose cervical count has so far been almost constant across taxa, with 9 to 10 vertebrae [7]. We initially considered the possibility for the short neck being an artifact of preservation. However, it is unlikely that the ribs have shifted along the vertebral column, given their almost constant spacing and association with the synapophysis in at least some vertebrae. Dermal shoulder elements may have been shifted during deposition but that does not affect the identification of the first dorsal vertebra, which is based on the rib morphology. Anteriorly, cervical vertebrae form a curved yet continuous string, making it unlikely for any vertebra to be dislocated. Thus, we accept the observed cervical count of six as an original anatomical feature.

With the addition of *Eohupehsuchus brevicollis*, Hupehsuchia now contains four monotypic genera. The number should in fact be five when counting the unnamed genus that was recognized by [4] based on IVPP V4070, which is clearly different from the four named genera, as the phylogenetic hypothesis witnesses (Fig. 6). The presence of five monotypic genera within a limited geographic range across about 80 km, only within the short time span of the latest Spathian, may appear excessive. However, these five genera are morphologically distinctive from each other, as demonstrated by the previous studies [4], [7], [8], as well as the present investigation. We find it difficult to reject the observed diversity based on morphological or phylogenetic reasons. Also, the five are divided into three size classes, with *Nanchangosaurus* and *Eohupehsuchus* being the smallest (about 40 cm in total length), *Hupehsuchus* and IVPP V4070 being intermediate at about 1 m, and *Parahupehsuchus* being the largest, probably approaching 2.0 m based on partial fossils. It is possible that body size differences helped them partition resource use in the region. Further paleoenvironmental studies are necessary to clarify this point.

The strange preservation of the left forelimb suggests that the tip of the limb was lost before the burial of the animal, and that the loss resulted in damage across the digits. Yet, this damaging mechanism only affected the tip of the left forelimb alone without affecting the rest of the animal. There are not many natural mechanisms that would cut off the tip of a paddle in this manner. The possibility always exists that the tip was caught between two rocks that cut it but such an accident is expected to be very rare, especially given that the preservational environment does not indicate a rocky bottom. A more plausible interpretation may be that the tip was bitten off by a vertebrate predator or scavenger. Given that the rest of the body is undisturbed, it is unlikely that a scavenger caused the damage. Also, no trace is present in the matrix surrounding the specimen to indicate the presence of invertebrate scavengers, whereas no fossil fish or an invertebrate scavenger is known from the Jialingjiang Formation. Then, the most likely cause would be a predator, from which the individual escaped after losing the tip of the left forelimb. If so, the bending and piling of broken phalanges would have been caused by the pressure from the teeth of the predator, which probably did not sharply cut off the tip in one bite. There is no evidence of healing, so the individual may not have lived for too long after it escaped. Given the small size of the injury, however, it is unlikely that the attack led to the eventual death unless the bleeding did not stop.

There is a record of sauropterygians in the Nanzhang-Yuan'an fauna that potentially fit the role of predator outlined in the scenario above. For example, *Hanosaurus hupehensis* [15] is a large pachypleurosaur [16] that is at least twice as large as *Eohupehsuchus*, unlike the other pachypleurosaur in the fauna, *Keichousaurus yuananensis* [17]. The type specimen of *H. hupehensis* has a maxilla that is 4.5 cm long, [15], [16]. The suspected injury in the present specimen is small, spanning slightly more than 1 cm (Fig. 2). Therefore, *H. hupehensis* is sufficiently large to have been able to cause the damage.

Predation pressure upon hupehsuchians was suggested previously based on anecdotal inferences [8]. The present specimen may add a more direct piece of evidence for the presence of predation pressure upon hupehsuchians, if the interpretation above is true. Possibly, the high predation pressure led to the quick diversification of hupehsuchians. However, it is difficult to test such a hypothesis.

## Supporting Information

S1 Dataset **Phylogenetic data matrix in NEXUS format.**
(NEX)

S1 Text **Phylogenetic data matrix and character descriptions.**
(DOCX)
