# Supplementary material for: A Small Short-Necked Hupehsuchian from the Lower Triassic of Hubei Province, China
Source: PLoS One. 2014 Dec 17;9(12):e115244. doi: 10.1371/journal.pone.0115244 (PMC4269458; doi:10.1371/journal.pone.0115244)
Supplement: S1 Text — Phylogenetic data matrix and character descriptions. (DOCX) [file pone.0115244.s002.docx]

**Text S1. Data matrix for phylogenetic analysis**

**Character Matrix**

**A NEXUS file containing the matrix is available as a separate file (DatasetS1.nex)**

Hovasaurus 00000000000000000000000000?0?000

Cartorhynchus 01011100?000100000?0100000?0?000

Chaohusaurus 0111000000(0 1)010001000101000?0?000

Nanchangosaurus 10(0 1)0110????????10110011011010100

Eohupehsuchus 1010101010010??00110111011010111

Hupehsuchus 10(0 1)010101(0 1)0101010111110011111110

Parahupehsuchus ?????0111111011?1111110111111111

IVPP.V4070 ??????111110011?111??101?????1?1

**Character Description**

(1) Snout, flattened: (0) FALSE; (1) TRUE.

(2) Nasal extended rostrally beyond external naris: (0) FALSE; (1) TRUE.

(3) Frontal, participation in orbital margin: (0) present; (1) absent.

(4) Large scleral ring filling the orbit: (0) absent; (1) present.

(5) Teeth: (0) small; (1) edentulous.

(6) Manual zeugopodials shortened: (0) FALSE; (1) TRUE.

(7) Carpal development: (0) delayed; (1) normal.

(8) Extra proximal carpal: (0) absent; (1) present.

(9) Radiale larger than other proximal carpals: (0) FALSE; (1) TRUE.

(10) Extra ant. distal carpal: (0) absent; (1) present.

(11) Extra anterior metapodial: (0) absent; (1) present.

(12) Digits 1-3 'bundled': (0) FALSE; (1) TRUE.

(13) Digital separation: (0) present at least partly; (1) absent.

(14) Manual digit 1 hyperphalangeal and with max number of phalanges: (0) FALSE; (1) TRUE.

(15) Extra proximal tarsal: (0) absent; (1) present.

(16) Cervical count: (0) 6 or less; (1) 9 or more.

(17) Dorsal count: (0) 29 or less; (1) 30 or more.

(18) Anterior dorsal neural spine, second segment: (0) absent; (1) present.

(19) Posterior dorsal neural spine, first segment, interspinal space: (0) present; (1) absent.

(20) Dorsal rib articulating with two vertebrae: (0) absent; (1) present.

(21) Parapophysis elevated above posterior dorsal centra margin: (0) absent; (1) present.

(22) Rib posterior flange: (0) absent; (1) present.

(23) Rib, pachyostosis midshaft: (0) present; (1) absent.

(24) Ribcage, depth: (0) swollen midway; (1) semi-constant.

(25) Gastralia, anterior flange overlapping anterior gastralia: (0) absent; (1) present.

(26) Lateral gastralia boomerang-shaped, pointing anteriorly: (0) absent; (1) present.

(27) Lateral gastral element each approximately symmetrical: (0) TRUE; (1) FALSE.

(28) Median gastralia v-shaped, pointing posteriorly: (0) absent; (1) present.

(29) Median gastralia cross-section: (0) flattened; (1) round.

(30) Dermal armor above dorsal neural spine, first layers: (0) absent; (1) present.

(31) Dermal armor above dorsal neural spine, third layer: (0) absent; (1) present.

(32) Dermal armor above caudal vertebrae with haemal arches: (0) absent; (1) present.

**Character correspondence**

| This paper | Ref. 8 |
| --- | --- |
| 1 | 1 |
| 2 | 2. modified |
| 3 | — |
| 4 | — |
| 5 | 3 |
| 6 | — |
| 7 | — |
| 8 | 8. modified |
| 9 | 9 |
| 10 | 10 |
| 11 | 11 |
| 12 | 12 |
| 13 | — |
| 14 | 13 |
| 15 | — |
| 16 | 14.modified |
| 17 | 14.modified |
| 18 | 15 |
| 19 | 16 |
| 20 | — |
| 21 | — |
| 22 | 17 |
| 23 | — |
| 24 | 18 |
| 25 | 19 |
| 26 | 20 |
| 27 | — |
| 28 | 21 |
| 29 | — |
| 30 | 23 |
| 31 | 24 |
| 32 | 25 |
| — | 4 |
| — | 5 |
| — | 6 |
| — | 7 |
| — | 22 |
